# The cellular basis of meristem development in fern gametophytes

**DOI:** 10.1042/BST20240728

**Published:** 2025-02-13

**Authors:** Chong Xie, Cankui Zhang, Xing Liu, Yun Zhou

**Affiliations:** 1 Department of Botany and Plant Pathology, Purdue University, West Lafayette, IN 47907, U.S.A; 2 Purdue Center for Plant Biology, Purdue University, West Lafayette, IN 47907, U.S.A; 3 Department of Agronomy, Purdue University, West Lafayette, IN 47907, U.S.A; 4 Department of Biochemistry, Purdue University, West Lafayette, IN 47907, U.S.A

**Keywords:** cell division, cell lineage, ferns, gametophytes, meristem, seed-free plants

## Abstract

The life cycle of land plants is characterized by alternating generations of sexual gametophytes and asexual sporophytes. Unlike seed plants, seed-free vascular plants, including ferns, initiate and maintain pluripotent meristems during their gametophyte phase to sustain body expansion and drive the formation of sexual organs for reproduction. This review summarizes meristem development among various fern species during the gametophyte phase, focusing on the cellular basis of meristem initiation, proliferation, and termination. We review the different types of gametophytic meristems in ferns, including apical cell (AC)-based meristems, multicellular apical meristems, and multicellular marginal meristems. We highlight both conserved and lineage-specific patterns of cell division, which are closely associated with these meristem identities and play crucial roles in shaping gametophytic morphology. Additionally, we highlight recent progress in understanding the dynamics of cell division and growth that drive meristem development, through studies that integrate confocal live imaging and computational quantitative analysis. Furthermore, we discuss the influence of environmental and genetic factors on cell division activity in fern gametophytes, including conserved transcriptional regulators that sustain meristem indeterminacy and proliferation in the model fern *Ceratopteris richardii*.

## Introduction

The life cycle of nearly all land plants, including both seed plants and seed-free plants, is characterized by an alternation of generations [1–4]. In this cycle, the plant alternates between a sexual gametophyte generation and an asexual sporophyte generation, ensuring continuous growth, development, and reproductive capacity [1–7]. Gametophytes produce gametes, which, upon fertilization, form a zygote that develops into a new sporophyte [6–10]. In seed plants, the sporophyte phase is dominant, while the gametophytes are significantly smaller in size, grow dependently on the sporophytes, and lack meristems [6,9,10]. In contrast, in ferns, which reproduce through spores rather than seeds, both gametophyte and sporophyte phases grow independently [7,11,12]. Fern gametophytes initiate and maintain pluripotent meristems that support prothallus expansion and drive the formation of sexual organs necessary for reproduction [8,13–16]. This review focuses on the dynamic development of meristems during the gametophyte phase across various fern taxa, including the model species *Ceratopteris richardii* (hereafter referred to as *Ceratopteris*). We summarize both conserved and species-specific patterns of cell division and growth that drive meristem development in fern gametophytes, with particular emphasis on recent findings from studies integrating time-lapse live imaging and computational image analysis. Additionally, we discuss the environmental and genetic factors that influence meristem development in fern gametophytes and highlight emerging key questions and new directions for future research in this field.

### Various types of meristems in fern gametophytes

After spore germination, fern gametophytes continually expand and dynamically change their shapes throughout their developmental processes [11,13–18]. At maturity, fern gametophytes across species also display a high degree of morphological complexity, with shapes including symmetric cordate (heart-shaped), asymmetric cordate, filamentous forms, and strap-like structures [11,13–18]. Similarly, gametophytic meristems display significant variability in morphology, cellular organization, and timing of cell proliferation [13,16]. The activity of meristems in gametophytes plays a significant role in shaping prothallus morphology. Based on their locations and patterns of cellular organization, various meristem identities can generally be categorized into three major types: apical cell (AC)-based meristems, multicellular apical meristems, and multicellular marginal meristems [13,16,19]. While the multicellular apical meristem and multicellular marginal meristem originate from different locations within the prothallus, they share similar features as multicellular meristems but remain morphologically distinct from AC-based meristems [19].

**Figure 1:**
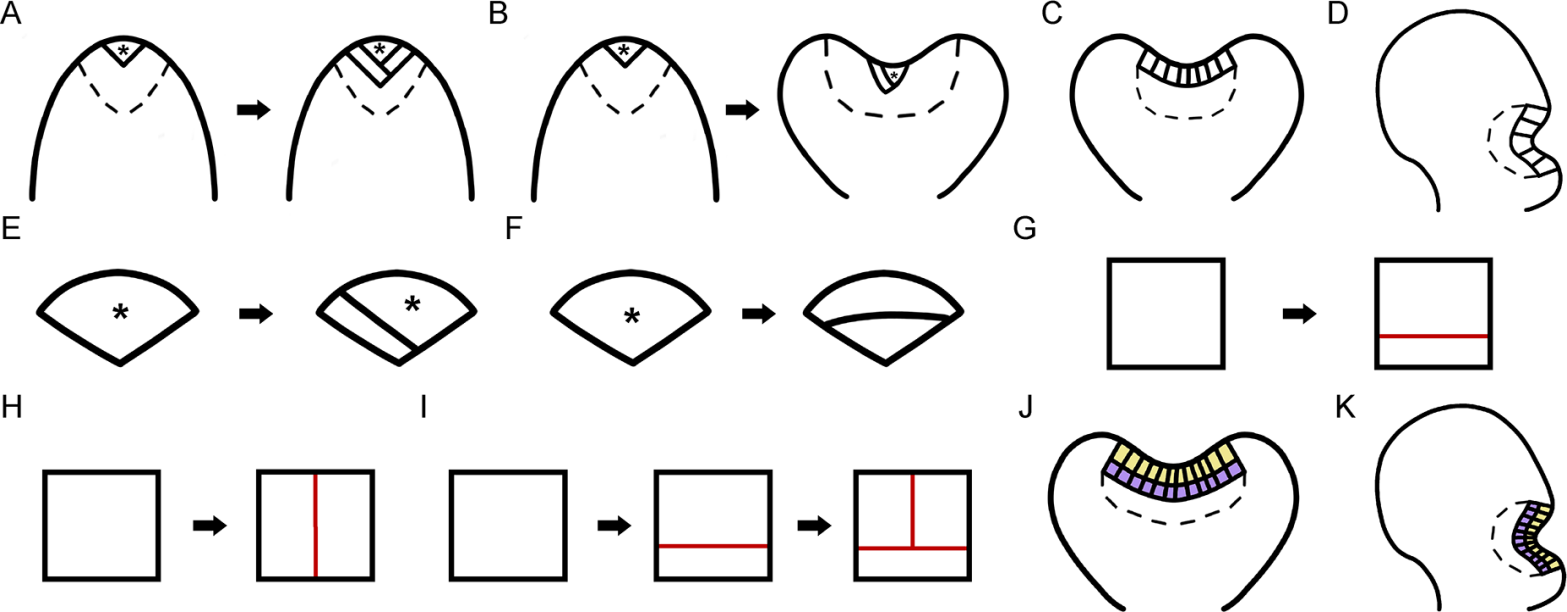
Diagrams illustrating cell division patterns in different meristems and cell layers of multicellular meristems in fern gametophytes. (**A–D**) Diagrams illustrating various types of meristems observed in fern gametophytes. (**A,B**) Wedge-shaped ACs (labelled with asterisks) and AC-based meristems. (**C,D**) Different locations of multicellular apical meristems and multicellular marginal meristems. (**E,F**) Types of cell divisions in ACs: the wedge-shaped AC either forms a new AC and a rectangular daughter cell (**E**) or undergoes a periclinal division to generate two daughter cells but losing its AC morphology (**F**). Asterisks indicate the ACs. (**G,H**) Two types of cell division in multicellular meristems: periclinal (**G**) and anticlinal divisions (**H**). (**I**) Reverse ‘T’ type cell division, with red indicating newly formed cell walls. (**J,K**) Illustrations of cell layers in multicellular meristems. Yellow: the outermost layer, purple: the second (inner) layer.

The AC-based meristem, composed of the iconic AC and its immediate progeny, drives the apical growth of fern gametophytes [16,19–22] (Fig. 1A,B). The AC, located at the prothallus apex, typically initiates early during gametophyte development following spore germination. Characterized as wedge-shaped or tetrahedral, the AC serves as the initial cell for proliferation, exhibiting division activity [16,19,23–26]. Through these divisions, ACs generate surrounding progeny while renewing themselves, thereby promoting gametophyte growth and expansion [16,19,23–26]. Notably, the duration of AC maintenance and the activity of the AC-based meristem vary significantly across fern species [16,19]. In *C. richardii* and *Anemia phyllitidis*, the wedge-shaped AC is only transiently present at the apex immediately after spore germination [8,27–32]. This transient AC quickly becomes morphologically indistinguishable as the prothallus expands, with the active proliferation site shifting to one lateral region of the prothallus, driven by the multicellular meristem [8,27–32].

Unlike transiently existed ACs, in the strap-shaped gametophytes of *Colysis decurrens*, a functional AC-based meristem is present at the apex immediately after spore germination [16,23]. The AC functions as the initial cell, producing 5–9 derivative cells before becoming morphologically indistinguishable and mitotically inactive [16,23]. Similarly, AC-based meristems in *Lygodium japonicum* and *Woodsia obtusa* maintain division activity during the early stages of gametophyte development [16,19,24,25]. These meristems typically undergo a few rounds of cell division before being directly replaced by multicellular apical meristems at the same apical region of the gametophytes [16,19,24,25]. In *W. obtusa* [25] and *L. japonicum* [24], the multicellular apical meristem is initiated with a row of several elongated, rectangular cells at the apex (Figure 1C), lacking any morphologically distinguishable AC. Once established, the multicellular apical meristem sustains active proliferation at the apex, driving prothallus expansion and the formation of the apical notch [16,19,24,25]. The difference between these two apical meristems—the AC-based meristem and multicellular apical meristem—aligns with the variable gametophyte morphology observed across fern taxa [16,19,24,25]. Studies in several different fern species have shown that the transition between these two types of apical meristems usually marks distinct stages of gametophyte development [16,19–25]. For instance, in *W. obtusa*, the AC-based meristem predominantly drives early prothallus growth, whereas the multicellular apical meristem becomes active during later developmental stages, promoting cell proliferation to establish an apical notch [25]. Interestingly, in several other species, such as *Sphenomeris chinensis*, *Blechnum australe*, and *Cyrtomium macrophyllum*, AC-based meristems persist throughout gametophyte development and directly contribute to the formation of the apical notch [19,26] (Figure 1B). In the gametophytes of these species, during the late developmental stages, the wedge-shaped AC retains its distinguishable morphology and division activity, and the AC-based meristem forms a deep notch at the center of the prothallus apex, separating the two fully expanded wings [19,26].

Unlike the AC-based meristem and the multicellular apical meristems discussed above, the multicellular marginal meristem [19,32]—also referred to as the marginal meristem [13,29], multicellular meristem [32,33], notch meristem [30,34], or lateral meristem [8,27,35] in earlier studies—is located and maintained on one lateral side of the prothallus (Figure 1D). This meristem consists of a row of adjacent, rectangular, actively dividing cells (Figure 1D), and its origin appears to be independent of AC activity. It plays a critical role in both the expansion of the prothallus and the initiation and development of reproductive organs [8,16,19,27,34,36]. For instance, in *A. phyllitidis*, the multicellular marginal meristem produces cells in both distal and proximal directions to form wings in both directions, resulting in an asymmetric, heart-shaped gametophyte [28]. In *Ceratopteris*, the multicellular marginal meristem drives the dynamic morphological changes of the gametophyte, ultimately leading to the formation of a heart-shaped, fully expanded prothallus at maturity [8,27]. It also continuously triggers the development of egg-bearing archegonia near the meristem notch prior to fertilization [8,27]. Additionally, in other fern species such as *Pteris vittata*, the multicellular marginal meristem and the AC can coexist but initiate at different regions of the same prothallus, with both sustaining cell division activity and promoting proliferation in different directions [31]. The co-existence of these two meristems, along with the variable timing of their initiation and termination, influences the shape of mature gametophytes and contributes to morphological variability observed within gametophyte populations [31]. This further supports the long-held view that meristem activity determines fern gametophyte morphology [13,14,16,31].

### Cell division patterns in fern gametophytes

In the meristems of fern gametophytes, cell divisions occur in various patterns, each directly associated with the initiation, maintenance, or termination of different meristem identities across fern taxa [13,16,19]. Specifically, in the AC-based meristem, the wedge-shaped AC undergoes an asymmetric division (Figure 1E) where a new cell wall unequally cut through its center, producing two daughter cells with distinct shapes: a new wedge-shaped AC and a flanking trapezoid-shaped cell [19,23–26,31]. This type of asymmetric cell division, commonly observed in AC-based meristems (Figure 1E), drives active cell proliferation and is closely linked to the initiation and self-renewal of ACs [19,23–26,31]. In several species, ACs remain mitotically active, continuously undergoing asymmetric divisions until the late stages of gametophyte development, primarily contributing to AC-mediated apical growth [26]. Specifically, the AC is divided into a new wedge-shaped cell and a large trapezoid-shaped cell, followed by periclinal division in the trapezoid-shaped cell, forming a cell packet at the notch center [26]. In contrast, in species such as *L. japonicum* and *W. obtusa*, after a few rounds of divisions that renew the AC, a periclinal division—where the cell divides parallel to the meristem margin—occurs in the newly formed wedge-shaped AC [24,25] (Figure 1F). This division (Figure 1F) results in the loss of the AC’s morphological signature, coinciding with the termination of the AC-based meristem and the subsequent transition to the multicellular apical meristem [24,25].

Multicellular meristem development involves both periclinal (parallel to the meristem margin, Figure 1G) and anticlinal (perpendicular to the meristem margin, Figure 1H) divisions (Figure 1G,H). During the initiation of the multicellular apical meristem, as observed in the gametophytes of *W. obtusa*, several anticlinal divisions occur simultaneously or sequentially at the apical center of the gametophyte, resulting in one row of adjacent rectangular cells [19,25] (Figure 1C). Following this, the proliferation and expansion of multicellular meristems, as seen in the gametophytes of *L. japonicum* and *W. obtusa*, are driven by the dynamic initiation and renewal of conserved three-celled rectangular packets [19,24,25]. These packets consist of one short rectangular cell at the base and two tall rectangular cells at the top (Figure 1I). The formation and renewal of these three-celled packets require a reverse-T type of cell division, which is conserved and widely observed across fern species [19,24,25]. This division type is named after the shape of the ﬁrst two dividing walls, which resembles a reversed “T” (Figure 1I). It involves two sequential divisions: one periclinal division in a rectangular cell, followed by an anticlinal division in the upper daughter cell (Figure 1I). Conversely, alternative combinations of anticlinal and periclinal divisions—such as an anticlinal division followed by a periclinal division in one upper rectangular cell, or two consecutive periclinal divisions in one upper rectangular cell—disrupt the formation of the three-celled packets, leading to their disappearance and the loss of their characteristic structure [19,25].

Additionally, during continuous proliferation, multicellular meristems gradually establish multiple cell layers, defined as the layers extending inward from the edge of gametophytes within the same two-dimensional plane (as illustrated in Figure 1J,K). These layers are typically characterized by the outermost, first cell layer (yellow in Figure 1J,K) and the second, inner layer (purple in Figure 1J,K). In *W. obtusa* gametophytes, anticlinal divisions occur with comparable frequency in both the outermost and inner layers of multicellular apical meristems, contributing to an increase in the cell number within each layer [25]. In contrast, periclinal divisions occur more frequently in the outermost layer than in inner cells, likely contributing to the formation of additional cell layers originating from the outermost layer within the multicellular meristems [25].

### Cell lineage dynamics, cell growth, and division activity during meristem initiation and maintenance in fern gametophytes

Recent advancements in genetic resources and techniques, including the establishment of stably transformed fluorescent reporters, noninvasive live-imaging platforms, and computational pipelines for image segmentation and quantification, have greatly expanded our knowledge of cell division and lineage dynamics during meristem initiation and maintenance in fern gametophytes [19,25,26,31,32]. For instance, quantitative analysis has shown that, in the gametophytes of several fern species, cell division activity is closely related to cell size and cell position [19,25,26,31]. Throughout the prothalli, cell divisions primarily occur in small cells, which are typically located at the apex or on one lateral side, corresponding to the multicellular apical meristem or multicellular marginal meristem [19,25]. The average size of dividing cells is significantly smaller than that of non-dividing cells [25]. In addition, in the gametophytes of ferns such as *W. obtusa*, the average cell expansion in dividing cells is moderately higher than that in non-dividing cells. This suggests that the smaller cell dimensions observed are not due to reduced cell expansion but rather increased division activity, a feature that appears to be conserved among many other meristems identified in seed plants [25].

**Figure 2:**
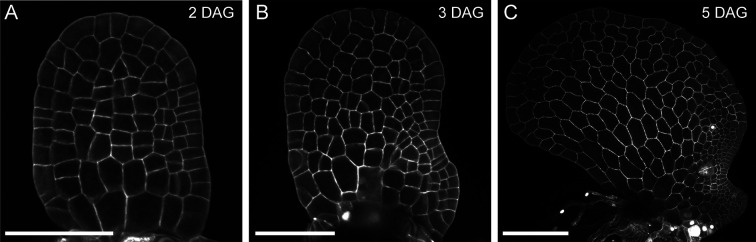
Confocal images of *Ceratopteris* hermaphroditic gametophytes at various days after germination (DAG). Different hermaphroditic gametophytes of *Ceratopteris* were stained with propidium iodide (PI) and imaged at 2 DAG (**A**), 3 DAG (**B**), and 5 DAG (**C**) using confocal microscopy. Gray: PI counterstain, highlighting cell outlines. Scale bar: 100 µm.

**Figure 3:**
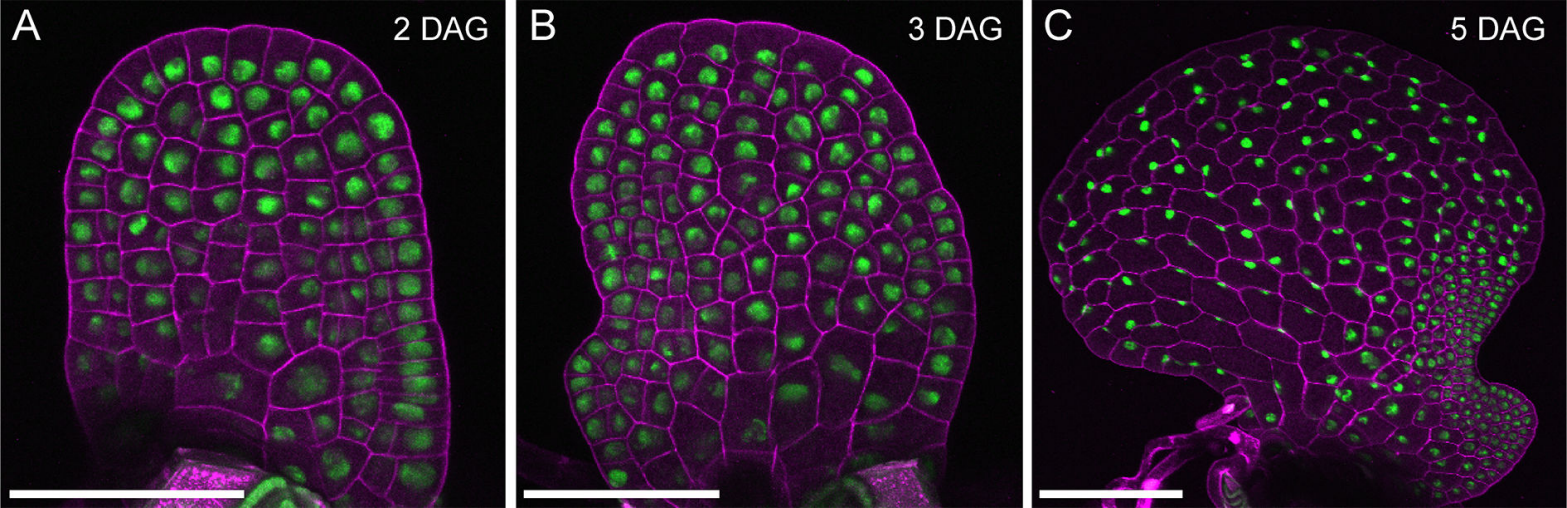
Confocal images of *Ceratopteris* hermaphroditic gametophytes expressing a fluorescent nuclear marker—*pCrHAM::H2B-GFP::3′CrHAM*. Different hermaphroditic gametophytes of *Ceratopteris* were stained with PI and imaged at 2 DAG (**A**), 3 DAG (**B**), and 5 DAG (**C**) using confocal microscopy. Green: GFP-labeled nuclei, magenta: PI counterstain showing cell outlines. Scale bar: 100 µm.

In *Ceratopteris* gametophytes, long-term time-lapse imaging of a fluorescent nuclear marker was performed during the initiation and proliferation of multicellular marginal meristems [32] (Figures 2 and 3). Using confocal live imaging [25,26,31–33] (Figures 2 and 3) and computer-assisted image analysis [25,26,31–33], meristem development was analyzed at single-cell (nucleus) resolution. Reconstructing cell lineage dynamics from an early stage of meristem development (starting with fewer than 40 cells in the gametophytes) reveals that the multicellular meristem originates from a few cells in the marginal layer [32]. These cells and their progeny undergo continuous proliferation, while other cell lineages lose division activity and only contribute to differentiated cells outside the meristem. Interestingly, within the meristem, cell division activity is independent of cell lineages but highly dependent on location, with marginal cells consistently maintaining higher division activity compared with inner cells [32]. These findings suggest a positional signal that dictates cell division and sustains meristem proliferation [32]. Additionally, the multicellular meristem in *Ceratopteris* gametophytes also induces adjacent cells to differentiate into egg-producing archegonia through position-dependent signaling [32].

### Regulators of cell division during meristem development in fern gametophytes

Since fern gametophytes are free-living, photosynthetic, and autotrophic, their cell division and growth are influenced by various external environmental factors [8]. Light intensity and quality are well-known signals that guide fern gametophyte development and can even influence the determination of different sex types [8,16,37–40]. In *Onoclea sensibilis*, continuous illumination leads to an increase in cell division rate and prothallus growth with higher light intensity, while the ratio of prothallus length to width decreases as light intensity increases [41,42]. In a few species, such as *Adiantum capillus-veneris*, red and blue lights regulate cell cycle progression differently during early prothallus expansion, resulting in distinct gametophyte morphologies under different light wavelengths [40–43]. Additionally, blue light induces the development of male gametophytes in *Ceratopteris*, which lack meristems, while red light appears to suppress the male differentiation program in *Ceratopteris* gametophytes [38].

In terms of internal cues, phytohormones such as auxins play crucial roles in regulating cell division and differentiation [42,44–47]. Exogenous auxin treatment assays suggest that auxins, including IAA, play a role in the development of multicellular marginal meristems in *Ceratopteris* gametophytes [44,46,47]. A previous study found that mechanical ablation of a few cells in the *Ceratopteris* multicellular meristem is sufﬁcient to induce new meristem regeneration outside the original meristem [32]. Interestingly, more recent work showed that auxin is involved in the regeneration of multicellular meristems after cell ablation, indicating its role in mediating cell proliferation in fern gametophytes [46]. Auxin also appears to facilitate communication between the marginal meristem and other regions of the developing gametophyte in *Ceratopteris* [44,46]. Additionally, in *O. sensibilis*, cells exhibit a more sensitive response to IAA when cell division occurs at shorter intervals, suggesting that this phytohormone might regulate cell division by affecting cell size [42]. Besides auxin, antheridiogen—a pheromone that determines sex types in fern gametophytes [8,27,39,48–50]—also plays a crucial role in meristem development and cell proliferation. In homosporous ferns, the absence of antheridiogen allows germinated spores to develop into meristic hermaphrodites, which possess a multicellular meristem. Conversely, in the presence of antheridiogen, germinated spores develop into ameristic male gametophytes, which lack meristems and instead differentiate to form numerous sperm-producing antheridia [8,27,34,36]. Antheridiogens in several fern species have been identified as gibberellins or gibberellin-like compounds [39,48,50], though the biochemical nature of antheridiogen in *Ceratopteris* remains unidentified. Interestingly, once a *Ceratopteris* hermaphrodite initiates a multicellular meristem, it begins secreting antheridiogen, which induces neighboring undetermined gametophytes to develop as male. At this point, the meristic hermaphrodite becomes insensitive to antheridiogen [27,50]. In contrast, *Ceratopteris* ameristic males do not produce antheridiogen but remain capable of sensing antheridiogen released by hermaphrodites [27,50]. These observations highlight a close relationship between meristem activity, antheridiogen production, and antheridiogen perception, deserving more studies in the future.

The HAIRY MERISTEM (HAM) family of GRAS-domain transcriptional regulators plays a crucial role in maintaining stem cell indeterminacy and proliferation in shoot apical meristems (SAMs) of seed plants during the sporophyte phase, including the model species *Arabidopsis thaliana* [51–58]. During the gametophyte phase of *Ceratopteris*, the *HAM* family gene *CrHAM* [33,57,59,60] interacts with the antheridiogen pathway to regulate sex differentiation and sustain meristem indeterminacy [33]. The CrHAM protein localizes within the multicellular meristem but is excluded from differentiated antheridia, consistent with its role in maintaining meristem cell proliferation [33]. Studies in both *Arabidopsis* and *Ceratopteris* have shown that HAM expression patterns in meristems are primarily determined by microRNA171 (miR171), and the signaling circuits centered on the miR171-HAM module appear to maintain conserved roles in both multicellular meristems of *Ceratopteris* gametophytes and SAMs of *Arabidopsis* sporophytes [33,51,52,55,57,61–63]. Additionally, a WUSCHEL-related homeobox (WOX) family gene *CrWOXB* is specifically expressed in the notch region of the multicellular meristem in *Ceratopteris* hermaphroditic gametophytes and in cells that have not yet differentiated into antheridia in male gametophytes. *CrWOXB* regulates meristem activity in *Ceratopteris* gametophytes, playing a key role in shaping prothallus morphology and influencing organ formation [35,64]. All these findings suggest the existence of largely unexplored yet exciting regulatory networks that connect various environmental signals and internal cues to sustain cell division and growth, as well as determine cell fate during meristem development in fern gametophytes.

### Concluding remarks

Gametophytic meristems in ferns, including the AC-based meristem, multicellular apical meristem, and multicellular marginal meristem, exhibit remarkable functional conservation and morphological diversity, playing pivotal roles in promoting prothallus expansion and sexual reproduction. This review highlights the conserved patterns of cell division associated with the initiation, maintenance, and termination of meristems, and emphasizes the division activity and lineage dynamics that sustain the function of meristems and shape the morphology of gametophytes. We also summarize the current understanding of the environmental signals and internal cues that regulate cell division and differentiation in fern gametophytes. In the future, the integration of molecular genetic resources and tools [12,33,65–69], transcriptomic studies [33,69], and live imaging approaches [25,32,70] will greatly help dissect the complex regulatory networks within meristems and gametophytes. It will be exciting to explore whether these regulators play conserved or diversified roles in meristem development and cell division in fern gametophytes, in comparison with their functions in other types of meristems in the sporophytes of seed plants. In addition, developing mathematical and computational approaches to more precisely determine and simulate cell division and growth will provide quantitative insights into meristem development.

**Perspectives**

- Meristems in free-living gametophytes of ferns offer unique research systems to address fundamental questions on stem cell behavior and homeostasis in multicellular organisms.
- Current studies that integrate confocal live imaging and computational image analysis are uncovering patterns of division activity and lineage dynamics during meristem development in fern gametophytes.
- Future research will further explore the molecular mechanisms underlying meristem cell proliferation in fern gametophyte by integrating molecular genetics, functional genomics, live cell imaging, and computational approaches.

## Abbreviations

AC: apical cell
DAG: days after germination
PI: propidium iodide
SAM: shoot apical meristem
miR171: microRNA171
